# Estimating the time of last drinking from blood ethyl glucuronide and ethyl sulphate concentrations

**DOI:** 10.1038/s41598-022-18527-8

**Published:** 2022-08-22

**Authors:** Lele Wang, Wei Zhang, Ruilong Wang, Yongli Guang, Daming Zhang, Chao Zhang, Meng Hu, Zhiwen Wei, Wenfang Zhang, Keming Yun, Zhongyuan Guo

**Affiliations:** 1 School of Forensic Medicine, Shanxi Medical University, Jinzhong, 030600 Shanxi China; 2 Key Laboratory of Forensic Toxicology of Ministry of Public Security, Jinzhong, 030600 Shanxi China; 3 Academy of National Food and Strategic Reserves Administration, Beijing, 100037 China; 4 Wanbailin District Public Security Bureau, Taiyuan, 030024 China; 5 Insititute of Forensic Science Tianjinn Binhai New Area Public Security Bureau, Tianjin, 300457 China

**Keywords:** Biomarkers, Medical research

## Abstract

The determination of length of time from the last drinking is critical for cases like drunk driving, sexual assault victims, and also postmortem suspected poisoning cases. The study was aimed to established a method of estimating the time of last drinking through the pharmacokinetic study of conjugation metabolites of alcohol in blood after a single oral dose. Twenty-six volunteers (14 males) consumed alcohol with food at a fixed dose of 0.72 g/kg after fasting for 12 h. Five milliliters of blood were collected 120 h after the start of drinking, and all samples were analyzed with headspace-gas chromatography and high-performance liquid chromatography–tandem mass spectrometry. The time point of last drinking was estimated through the relationship between the concentration ratio of ethyl glucuronide to ethyl sulphate and the length of time after drinking. Pharmacokinetic parameters were analyzed by a pharmacokinetic software DAS according to the non-compartment model. A good correlation model was obtained from the relationship between concentration ratio of ethyl glucuronide to ethyl sulphate in blood and the time of alcohol use, and the margin of error was mostly lower than 10%. The time of maximum concentration, maximum concentration, and elimination half-life of ethyl glucuronide in blood were 4.12 ± 1.07 h, 0.31 ± 0.11 mg/L and 2.56 ± 0.89 h; the time of maximum concentration, maximum concentration, and elimination half-life of ethyl sulphate in blood were 3.02 ± 0.70 h, 0.17 ± 0.04 mg/L, and 2.04 ± 0.76 h. The study established a potential method to estimate the length of time after a moderate oral dose, and provided pharmacokinetic parameters of ethyl glucuronide and ethyl sulphate in Chinese population.

## Introduction

As a psychoactive substance with dependence-producing properties, alcohol has been one of the most widely abused drugs in the world. Due to the high prevalence of alcohol use in the general population, the number of alcohol-related accidents is increasing worldwide on the road, at work, and during acts of violence^1^, and the traffic accidents are the most common cases^2^. In most cases, however, we can no longer detect alcohol 6–8 h after drinking because of its rapid metabolism^3^, which makes it more complex to confirm drunk driving in a suspected hit-and-run accident^4^.

Alcohol is metabolized mainly through liver, and oxidized by alcohol dehydrogenases and aldehyde dehydrogenases eventually to acetyl CoA. Besides, there are other non-oxidative pathways of alcohol metabolism, such as conjugation reactions and fatty acyl synthases^5^. Ethyl glucuronide (EtG) and ethyl sulphate (EtS) are two major non-oxidative metabolites of alcohol produced by glucuronidation and sulfonation of alcohol. A very small amount (less than 1%) of the ethanol dose consumed is excreted in the urine as EtG and EtS^6^, which can be detected in many body fluids and tissues for an extended time after alcohol is eliminated from the body^7^. Therefore, EtG and EtS are expected to be sensitive and specific biomarkers for alcohol intake^8–10^.

The determination of recent alcohol use is critical for forensic purposes to evaluate cases such as drunk driving, sexual assault victims, and also postmortem suspected poisoning cases. The length of time from the last drinking is an important clue in some cases because it determines the nature of the case to some extent. Some methods have been reported to predict the time of last drinking^10,11^, and the concentration ratios of parent to its metabolites or the ratios of different metabolites were adopted in time estimation^12,13^, since the calculation of ratios can counteract the influence of dosage. However, the alcohol concentration is usually detectable within 8 h after ingestion, and considered accurate when measured within 24 h after death and at a temperature lower than 20 °C^14^. Therefore, we must be careful with the application of alcohol concentration in the analysis of cases, and a more reliable method is needed to estimate the time of last drinking. As the non-oxidative metabolites of alcohol, EtG and EtS have not only been reported higher concentrations and longer detection windows, which enable the retrospective assessment of alcohol intake even when alcohol itself was no longer present in the body^15,16^, but have been proved that they can not be produced after death^9,14^, and are stable at low temperature^17^. Consequently, the concentration ratio of EtG to EtS can be considered to estimate the time of last drinking.

The study aimed to established a method of estimating the time of last drinking through the pharmacokinetic study of EtG and EtS. Blood is the most commonly used matrix in alcohol-related cases. Compared with urine, analyte concentration in blood is rarely affected by the amount of water consumed, and its collection can not be easy to fake as well. Therefore, in this study, we investigated the pharmacokinetics of EtG and EtS in blood of 26 volunteers following a single dose of 0.72 g alcohol/kg to estimate the time of last drinking.

Pharmacokinetics of alcohol and its metabolites, especially the conjugation metabolites, are useful for the detection of alcohol consumption or relapse of alcoholics. Although that EtG and EtS have been reported longer detection windows, few studies about the pharmacokinetic parameters have been calculated^9,18^. The study is not only expected to establish a method of estimating the length of time after drinking, but also can provide pharmacokinetic parameters of EtG and EtS in Chinese population after a moderate oral dose.

## Materials and methods

### Chemicals and reagents

Alcohol (10 mg/mL) was obtained from AccuStandard, USA. Tertiary butanol (AR, ≥ 99.0%) was obtained from Aladdin, Shanghai. EtG (100 mg/mL) and two internal standards (IS, EtG-D_5_ 1 mg/mL and EtS-D_5_ 1 mg/mL) were obtained from Cerilliant, USA; EtS-Na (98%) was purchased from TSI, Japan. Methanol (HPLC grade) and acetonitrile (HPLC grade) were from Merke, USA; formic acid (LC/MS grade) was from J&K Chemical, China; ultrapure water was obtained from a water purification system (Milli-Q Academic, USA).

### Participants and study design

A total of 26 adults, including 14 males and 12 females without histories of somatic or psychiatric illness, drinking, or medication use, participated in the study and provided informed consent to the Committee of Medical Ethics of Shanxi Medical University (2018LL349). Their median age is 24.5 years (ranging from 22 to 27) and the average body mass index is 20.9 kg/m^2^ (ranging from 16.8 to 34.6). All participants resided in a secure clinical research unit for at least 24 h afterward, and they were medically evaluated during alcohol administration and 3 days later for the sake of safety. All participants got paid for their time and efforts.

After 12 h of fasting, the participants received Fenjiu, a local liquor containing 40% alcohol, proportionately to their weight (0.72 g/kg) accompanied by food within 30 min. Five millilitres of blood were drawn each time from an indwelling venous catheter in the arm before drinking and at the time points of 0.5, 1.5, 2, 3, 5, 8, 12, 24, 36, 48, 120 h after the start of drinking, respectively. All samples were stored at − 20 °C until analysis.

### Sample preparation

#### Alcohol

The preparation and detection methods were performed according to those used in our preliminary research^19^, and alcohol concentration was determined via the internal standard method using headspace-gas chromatography, with which tertiary butanol was the internal standard.

Briefly, 1 mL blood and 1 mL tertiary butanol (IS, 87 mg/mL) were added in a headspace bottle, next, the mixture was sealed after being diluted with 3 mL ultrapure water and then analyzed with headspace-gas chromatography.

#### EtG and EtS

Two internal standards (EtG-D_5_ and EtS-D_5_, 1 µg/mL, 100 µL) were added to 100 µL of blood to improve the identification and quantification of the metabolites, EtG and EtS. Proteins were precipitated with 800 µL of 80% acetonitrile in methanol for 10 min at 0 °C. After centrifugation (13,000 rpm, 5 min), the supernatant was removed and dried at 35 °C with nitrogen. The residues were redissolved with 400 µL of 5% acetonitrile in water, and centrifuged again at 13,000 rpm for 5 min. From the newly prepared solution, 3 µL was taken and injected into Liquid Chromatography tandem mass spectrometry (LC–MS/MS) for analysis.

### LC–MS/MS analysis

Chromatographic separation was performed with an Inertsil ODS-3 column (2.1 × 100 mm, 3 µm) through a LC-20A system (Shimadzu, Japan). The mobile phase was a mixture of solvent A (0.1% formic acid in ultrapure water) and solvent B (0.1% formic acid in acetonitrile). The chromatographic column was held at 35 °C and eluted for 14.0 min in total at a flow rate of 0.2 mL/min with a gradient of 5% B (0–2.0 min), 90% B (2.0–6.0 min), 90% B (6.0–8.0 min), 5% (8.0–8.5 min), and 5% (8.5–14.0 min), respectively. Mass spectrometry was performed on a mass spectrometer (TRAP 4000, Sciex, AB). All analytes were ionized by electrospray in negative mode, and tandem MS analysis was performed in multiple reaction monitoring (MRM) mode. The ionspray voltage was − 4000 V, and the temperature was 500 °C. The curtain gas, Gas1, and Gas2 were 40 psi, 50 psi, and 35 psi, respectively. Other specific MRM parameters for each analyte are shown as follows in Table 1.

**Table 1:** The feature ion pair and mass spectrum data of each analyte.

Analyte	Qualitative ions (m/z)	Quantitative ions (m/z)	DP (V)	CE (V)
EtS	125.0/80.0	125.0/97.0	46	45
	125.0/97.0			21
EtG	221.1/75.0	221.1/75.0	63	22
	221.1/85.0			23
EtG-D5	226.1/75.0	226.1/75.0	63	23
	226.1/85.0			26
EtS-D5	130.0/80.0	130.0/80.0	46	46
	130.0/97.9			25

### Estimation of the time of last drinking

The current study used the relationship between concentration ratios of EtG to EtS in blood and time to estimate the time of last drinking, and the errors between observed and actual time of last drinking were calculated with (|observed value − actual value|/actual value) × 100%.

### Statistics

The pharmacokinetics parameters were calculated with the software of DAS 3.0 according to the non-compartmental method. All data were summarized with descriptive statistics. Arithmetic means and standard deviations of key data (concentrations and detection time points) and pharmacokinetic parameters were provided. All statistical analyses were performed with version 13.0 of IBM SPSS^®^ Software (SPSS Inc., Chicago, IL, USA).

### Ethics approval

All experiments were performed in accordance with the current China legislation and approved by the Committee of Medical Ethics of Shanxi Medical University (2018LL349).

### Informed consent

All subjects provided written informed consent prior to screening.

## Results

### Method validation

All analytes including alcohol, EtG, EtS, EtG-D_5_ and EtS-D_5_ were well separated, no endogenous peak was observed to be coeluted with analytes, and the methods were fully validated as shown in Tables 2 and 3. The limit of detection (LOD) and limit of quantitation (LOQ) of EtG and EtS in blood were 0.02 µg/mL and 0.05 µg/mL, respectively. We redissolved the residues with 100 µL of 5% acetonitrile in water to quantitate the concentrations below the LOQ.

**Table 2:** Linearity range and detection limit of EtG and EtS in blood.

Analyte	linearity range (µg/mL)	linearity equation	R^2^	Weighting	LOD (µg/mL)	LOQ (µg/mL)
EtG	0.05–5.0	y = 1.7411x + 0.0036	0.9999	None	0.02	0.05
EtS	0.05–5.0	y = 1.803x − 0.0279	0.9997	None	0.02	0.05

**Table 3:** The precision, recovery, and matrix effect of EtG, EtS in blood.

Analyte	Concentration (µg/mL)	Intra-precision (%)	Inter-precision (%)	Recovery (%)	Matrix effect (%)
EtG	0.05	5.2	4.8	70.1	15.4
	0.5	4.8	2.4	66.9	11.5
	5.0	4.7	2.0	66.1	11.9
EtS	0.05	4.0	5.7	85.1	3.9
	0.5	4.7	2.0	82.0	1.0
	5.0	2.5	6.1	88.6	0.9

### Estimation of the time of last drinking

Based on the mean concentrations of EtG and EtS in blood showed in Table 4, we calculated the mean concentration ratios of EtG to EtS, and analyzed the relationships between the ratios and last usage after single oral dose. It was found that the interval time only correlated well using binomial function (y = 1.646x^2^ − 0.9599x + 0.0878, x represented ratio, y represented time, R^2^ = 0.9904), and the errors between observed and actual values [(|observed value − actual value|/actual value) × 100%] were mostly lower than 10% within 8 h, as shown in Table 7.

**Table 4:** The mean concentrations of alcohol and its metabolites in human blood ($\overline{{\text{x}}}$ ± S (min–max), n = 26).

Time	BAC (mg/mL)	EtG (μg/mL)	EtS (μg/mL)
0	–	–	–
0.5 h	0.34 ± 0.10 (0.19–0.61)	0.05 ± 0.02 (0.02–0.08)	0.06 ± 0.02 (0.03–0.09)
1.5 h	0.41 ± 0.11 (0.22–0.66)	0.14 ± 0.04 (0.06–0.24)	0.11 ± 0.02 (0.06–0.16)
2 h	0.41 ± 0.12 (0.14–0.60)	0.20 ± 0.06 (0.10–0.34)	0.14 ± 0.03 (0.07–0.20)
3 h	0.36 ± 0.14 (0.07–0.63)	0.27 ± 0.09 (0.13–0.47)	0.16 ± 0.04 (0.07–0.25)
5 h	0.17 ± 0.10 (0.00–0.37)	0.29 ± 0.12 (0.09–0.53)	0.14 ± 0.05 (0.03–0.24)
8 h	0.03 ± 0.04 (0.00–0.14)	0.14 ± 0.08 (0.04–0.30)	0.06 ± 0.03 (0.01–0.11)
12 h	–	0.04 ± 0.03 (0.01–0.12)	0.02 ± 0.01 (0.00–0.04)

Alcohol, EtG and EtS were not detectable at 24 h, 36 h, 48 h and 120 h after ingestion.

– not detectable; *BAC* blood alcohol concentration; all values were reserved for two decimal places.

### Pharmacokinetic analysis

**Figure 1:**
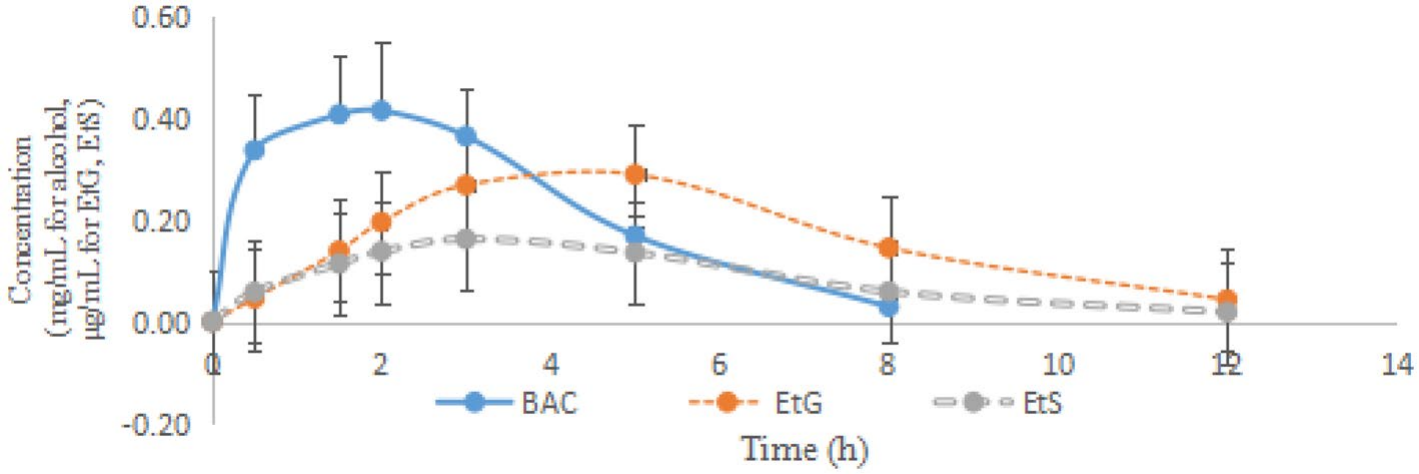
The mean concentration–time curve of alcohol, EtG and EtS in the blood.

Data on concentrations at each time point and detection limits of alcohol and EtG, EtS are shown in Tables 4 and 5. It was showed that after the volunteers’ consumption of 0.72 g alcohol/kg, alcohol concentration reached the highest level of 0.41 ± 0.11 mg/mL at 1.5 h and then declined gradually, and the detection window time was 3–8 h. The peak blood levels (maximum observed value) of EtG (0.29 ± 0.12 µg/mL) and EtS (0.16 ± 0.04 µg/mL) were observed at 5 h and 3 h, respectively, and the detection window were 8–12 h and 5–12 h. Additionally, as shown in Fig. 1, it was found that the concentrations of EtG were always higher than those of EtS through the process of study.

**Table 5:** The detection time of alcohol, EtG and EtS in blood ($\overline{{\text{x}}}$ ± S (min–max), n = 26).

Alcohol (h)	EtG (h)	EtS (h)
5.81 ± 1.74 (3.00–8.00)	22.15 ± 4.42 (12.00–24.00)	16.92 ± 6.23 (8.00–24.00)

Based on the non-compartment model, the pharmacokinetic parameters of alcohol and EtG, EtS after oral administration of 0.72 g alcohol/kg were calculated and are shown in Table 6. It was found that alcohol could reach its peak concentration in blood (441.65 ± 113.86 mg/L (0.44 ± 0.11 mg/mL)) at 2.02 ± 0.54 h, and the metabolites, EtG and EtS reached their peak concentrations (obtained from the pharmacokinetic model) (0.31 ± 0.11 mg/L and 0.17 ± 0.04 mg/L) at 4.12 ± 1.07 h and 3.02 ± 0.70 h, respectively. The t1/2z of alcohol and EtG, EtS were 1.24 ± 1.09 h and 2.56 ± 0.89 h, 2.04 ± 0.76 h, respectively. The CLz/F of alcohol was 0.49 ± 0.33 L/h; however, CLz/F of the metabolites of alcohol (EtG and EtS) could not been calculated accurately because their initial dose was different to estimate.

**Table 6:** Pharmacokinetic parameters of alcohol and its metabolites in human blood ($\overline{{\text{x}}}$ ± S, min–max, n = 26).

Parameter	Alcohol	EtG	EtS
AUC(0–t) (mg/L*)	1715.23 ± 626.72 (505.00–2914.00)	1.99 ± 0.78 (0.76–3.55)	1.04 ± 0.34 (0.41–1.75)
t1/2z (h)	1.24 ± 1.09 (0.30–4.23)	2.56 ± 0.89 (1.03–4.94)	2.04 ± 0.76 (1.11–3.32)
T_max_ (h)	2.02 ± 0.54 (1.50–3.00)	4.12 ± 1.07 (2.00–5.00)	3.02 ± 0.70 (1.50–5.00)
C_max_ (mg/L)	441.65 ± 113.86 (238.20–656.00)	0.31 ± 0.11 (0.13–0.53)	0.17 ± 0.04 (0.08–0.28)
Vz/F (L/kg)	0.69 ± 0.49 (0.11–1.76)	–	–
CL_Z_/F (L/h)	0.49 ± 0.33 (0.17–0.43)	–	–

## Discussion

The analytical method developed in this study has proved to be sensitive and accurate for the analysis of EtG and EtS. The lower LOD (0.02 µg/mL) and LOQ (0.05 µg/mL) achieved are particularly useful for quantifying the lower concentrations of EtG and EtS present in blood during pharmacokinetic investigation of EtG and EtS. Also, it is illegal in China to drive with a blood alcohol concentration (BAC) higher than 0.2 mg/mL, and C_max_ of BAC (0.22–0.66 mg/mL) at 0.72 g/kg dose of alcohol used in the study is close to that, suggesting that the method might be suitable for monitoring most cases of drunk driving in China.

This study has focused on the concentration–time-curves of alcohol, EtG and EtS in blood of 26 volunteers following a single dose of 0.72 g alcohol/kg, and aimed to establish a method of estimating the time of last drinking. It was found that the time of post-alcohol use could be estimated using the relative concentrations of EtG and EtS. In the preciously studies, Zhao et al.^10^ once proposed predicting the time of last drinking using the serum concentration ratio of alcohol to EtG. While alcohol is usually metabolized quickly in living body, and the alcohol concentration after death is subject to change due to postmortem redistribution^20^ and postmortem production^21^. Therefore, the application of concentration ratios of alcohol to metabolites has great limitations, and as reported in the literature, our study also showed a poor correlation between the concentration ratio of alcohol to its metabolites and time of last drinking. While, despite great individual differences, a good correlation model was obtained from the mean concentration ratio of EtG to EtS in blood and the time of alcohol use, and most of the prediction errors were less than 10%. This would be useful in forensic investigation such as in the case of driving under the influence of alcohol.

Pharmacokinetic parameters of alcohol and EtG, EtS in blood were calculated based on the non-compartment model. For alcohol, the study found that it reached the C_max_ of 441.65 ± 113.86 mg/L (0.44 ± 0.11 mg/mL) at 2.02 ± 0.54 h, and compared with the previous studies^11^, it showed a longer absorption phase. In addition, we found that alcohol in blood of the participants could be detected within 8 h (3–8 h), and the mean elimination half-life of alcohol was 1.24 ± 1.09 h (0.30–4.23 h), with individual differences. For EtG and EtS, in view of their longer detection window, we investigated the concentrations of analytes in blood up to 120 h after drinking, and it was the first time that pharmacokinetics of EtG and EtS in blood were evaluated in a larger number of Chinese population. Compared with EtS, EtG was discovered early and has been most studied^10,22–24^. It was reported that the detection windows of EtG in both serum and urine were longer than those of alcohol^25–27^, and we confirmed that EtG did metabolize slower than alcohol, and the elimination half-life of EtG in blood was 2.56 ± 0.89 h. Additionally, the detection time limit for urinary EtG was reported to depend weakly on the breath ethanol concentrations^27^, and our study showed no correlation between initial alcohol concentrations and detection limits of EtG, based on the CORREL function, suggesting that the lower alcohol consumption might not affect the detection limit of EtG. EtS was another non-oxidative metabolite of alcohol, and its concentration–time curve was found similar to that of EtG^8,18^. Georg Schmitt et al.^18^ once reported that the C_max_ of EtS ranged from 0.09 µg/mL to 0.72 µg/mL at 0.52 ± 0.17 g/kg dosage of alcohol, and the T_max_ was about 2.94 h, while in our study, the C_max_ of EtS was 0.17 µg/mL (ranging from 0.08 µg/mL to 0.28 µg/mL) at 0.72 g/kg dosage, and the T_max_ was 3.02 h, indicating interethnic differences. In addition, our study found that both the detection window and C_max_ of EtS were lower than those of EtG. However, EtS has been reported to be stable and not susceptible to bacteria^28,29^, suggesting that EtS could provide complementary data in the identification of alcohol ingestion.

Alcohol metabolism is influenced by many factors including the amount of alcohol consumption, age, gender, race, and genetic variability^30^, among which genetic difference between individuals has been considered a major contributor to the pharmacokinetic variation of alcohol. Gudrun^31^ and Lostia^32^ reported that the concentrations of alcohol, EtG, and EtS would increase as the dosage of alcohol intake increases, and differ in individuals with the same dosage of alcohol. A homogenous sample was obtained in our study, since the 26 Chinese Han people recruited were young and healthy, and all of them were free from somatic or psychiatric illness, alcohol drinking, and regular medication. Although effects of these parameters can not be ruled out, the results obtained in this study do suggest the association of the diversified pharmacokinetics of alcohol in Chinese population with genetic difference between individuals. To further elucidate the mechanism underlying the observed differences, we need to investigate the metabolic enzymes phenotyping of the 26 Chinese participants in future studies.

Race is another important element affecting the result of alcohol pharmacokinetics. Alcohol dehydrogenase 2 (ADH2), aldehyde dehydrogenase 2 (ALDH2) and CYP2E1 are three major ethanol-metabolizing enzymes. It was reported that the allele frequencies in Chinese populations (76.7%, 15.6%, and 28.9% respectively) were obsevably higher than those in European groups (0%, 0%, and 5.1% respectively)^33^, and a strongly protective variant in ALDH2 is essentially only found in Asians^34^. Uridine diphosphate-glucuronosyltransferases (UGT) and sulfotransferases (SULT) are two enzymes responsible for EtG and EtS formation. It was reported that genes coding for these enzymes have considerable polymorphism^35^, while few studies reported the interethnic difference of the allele frequencies of UGT and SULT. Apart from the dosage, here results showed a possible influence of race on the formation of EtG and EtS. Further studies on the influence of race on the glucuronidation and sulfonation of alcohol should be considered in the next studies.

Factors affecting the metabolism of alcohol might affect the ratio of EtG/EtS, and then affect the outcome of time estimation. In this study, we estimated the drinking time of volunteers based on the individual value of EtG/EtS at each time point (data was not showed here), and it was found that there was still great individual difference, especially for the application of extreme values like min and max EtG/EtS. Generally, the calculation of ratios can counteract the influence of dosage, but this is not the case when the dosage is too large, which is due to the report that sulphation is more saturable than glucuronidation at higher concentrations of substrate. And our study also showed an influence of dosage on the time estimation, especially when the metabolic ability was also relatively poor or strong. As shown in Table 7, the min values of observed time were obtained from a volunteer with the lowest dosage of alcohol due to his lighter weight, at the same time, the volunteer had not only a poor ability of alcohol metabolism (t_max_ = 3 h), but a lower level of EtG (C_max_ = 0.18 μg/mL) and higher level of EtS (C_max_ = 0.20 μg/mL). Conversely, this was also true for the max values of observed time. Here the estimated model to calculate the period of time after drinking was only based on a unique dosage of 0.72 g/kg, and the facts affecting the metabolism of alcohol, such as genotypes of metabolic enzyme, were also not considered, therefore, further studies on the influence of these factors affecting alcohol metabolism on the time estimation could also be needed.

**Table 7:** The errors between the time deduced from the formula and actual time of last drinking.

EtG/EtS (min–max)	Observed (h) (CI, min–max)	Actual (h)	Error (%)
0.00	0.00	0.00	0.00
0.79 (0.32–1.29)	0.35 (0.27–0.63, − 0.05 to 1.58)	0.50	29.05
1.22 (0.48–1.91)	1.36 (1.16–2.04, 0.01 to 4.28)	1.50	9.06
1.42 (0.59–2.44)	2.03 (1.64–3.02, 0.09 to 6.17)	2.00	1.41
1.65 (0.75–2.75)	2.99 (2.68–4.86, 0.29 to 9.93)	3.00	0.18
2.13 (0.94–3.56)	5.53 (5.04–8.43, 0.65 to 17.54)	5.00	10.54
2.45 (1.08–4.26)	7.64 (6.57–11.52, 0.96 to 25.89)	8.00	4.46

*CI* confidence interval (95%).

Additionally, cumulative effects caused by drinking cumulatively can also affect the calculation of last drinking. Here we simulated the drinking pattern at a normal meal of Chinese, namely, all volunteers were required to finish drinking within half an hour. In fact, drinking back and forth for more than half an hour is a rather normative drinking situation for most recreational alcohol drinkers. In this condition, there should be a cumulative addition of EtG and EtS concentrations in blood originating from these different drinking periods, and it is not possible to use the proposed model. Therefore, more studies on the impact of this for the calculation of last drinking should be also done in the next step.

In summary, the current study established a method of estimating the time of last drinking using the relationship between concentration ratio of EtG to EtS in blood and the time of alcohol use, and once further validated, this novel discovery will provide a useful analytical tool for monitoring alcohol use by Chinese motorists on the road. Additionally, we investigated the pharmacokinetics of alcohol and EtG, EtS in blood of Chinese population, and obtained the pharmacokinetic parameters of the targets. The sensitive LC–MS/MS approach developed and validated in the study can be applied in drink driving and other forensic cases when alcohol is involved, and the long detection windows of EtG and EtS support their use as useful markers for the detection of alcohol consumption.

## Data Availability

All data generated during the study appear in the submitted article.
